# *%polynova_2way*: A SAS macro for implementation of mixed models for metabolomics data

**DOI:** 10.1371/journal.pone.0244013

**Published:** 2020-12-15

**Authors:** Rodrigo Manjarin, Magdalena A. Maj, Michael R. La Frano, Hunter Glanz

**Affiliations:** 1 Department of Animal Science; California Polytechnic State University, San Luis Obispo, California, United States of America; 2 Department of Biological Sciences, California Polytechnic State University, San Luis Obispo, California, United States of America; 3 Center for Applications in Biotechnology, California Polytechnic State University, San Luis Obispo, California, United States of America; 4 Department of Food Science and Nutrition, California Polytechnic State University, San Luis Obispo, California, United States of America; 5 Center for Health Research, California Polytechnic State University, San Luis Obispo, California, United States of America; 6 Department of Statistics, California Polytechnic State University, San Luis Obispo, California, United States of America; CIC bioGUNE, SPAIN

## Abstract

The generation of large metabolomic data sets has created a high demand for software that can fit statistical models to one-metabolite-at-a-time on hundreds of metabolites. We provide the ***%polynova_2way*** macro in SAS to identify metabolites differentially expressed in study designs with a two-way factorial treatment and hierarchical design structure. For each metabolite, the macro calculates the least squares means using a linear mixed model with fixed and random effects, runs a 2-way ANOVA, corrects the P-values for the number of metabolites using the false discovery rate or Bonferroni procedure, and calculate the P-value for the least squares mean differences for each metabolite. Finally, the ***%polynova_2way*** macro outputs a table in excel format that combines all the results to facilitate the identification of significant metabolites for each factor. The macro code is freely available in the Supporting Information.

## Introduction

Metabolomics is the broad scale analysis of compounds involved in metabolism, primarily utilizing liquid and gas chromatography mass spectrometry, and nuclear magnetic resonance [1]. Matrices analyzed range from plants and cell media to animal and human tissues and fluids. Often capturing hundreds of metabolites from multiple metabolic pathways in one analysis, it is ideal for hypothesis generation. This is particularly true for untargeted metabolomics which typically attempt to analyze as many metabolites as possible to produce peak area or peak height data [2] that is often not calibrated by standards. Typically, the possible chemical identity of metabolites is not known before data are acquired, requiring post data-acquisition identification. This analysis can capture amino acids, sugars, metabolic pathway intermediates, and fatty acids, among many others. Global lipidomics assays are often untargeted and captures a variety of complex lipids including phospholipids, triacylglycerols, and cholesterol esters. Targeted metabolomics analyzes a large number of metabolites but screens for specific classes of metabolites and utilizes calibration standards for each metabolite, in addition to internal standards, to generate quantitative data. Lastly, semi-targeted metabolomics, a hybrid of untargeted and targeted metabolomics, screens for metabolites from several classes whose chemical identity is known prior to data acquisition and produces data in which one calibration curve and internal standard is used to quantify several metabolites.

The generation of these large data sets consisting of hundreds of metabolites has created a high demand for software tools capable of streamlining implementation of sophisticated methods of data analysis for a variety of study designs. Furthermore, the concern for the increased type one error associated with multiple hypothesis testing also requires the utilization of Bonferroni or false discovery rate (FDR) correction [3,4]. Various software packages have been created to enable more efficient statistical analysis of large data sets such as MetaboAnalyst and Metabox [5,6]. As a result, researchers without a programming background can easily conduct multivariate tests on hundreds of metabolites. However, these programs have numerous limitations. More specifically, to our knowledge a software is not available that can conduct analysis of variance (ANOVA) and generate FDR or Bonferroni-adjusted P-values for the selection of metabolites in study designs with a factorial treatment and hierarchical design structure. This is a particular concern since metabolomics is increasingly utilized in complex studies that require advanced statistical modelling to capture all sources of variability. This article details the key aspects and validation of a macro written in SAS software capable of modelling fixed and random effects in multifactorial ANOVA for large metabolomic datasets.

## Materials and methods

### Experimental data collection

An experimental dataset from a previously published study in our lab [7] was used to illustrate the utilization of the ***%polynova_2way*** SAS macro. Data is provided in S1 Data file. Experiments were conducted in accordance with the Institutional Animal Care and Use Committee of California State University (Protocol #1611 approved on 9/13/2017), and the National Research Council Guide for the Care and Use of Laboratory Animals. Animals were euthanized using an intramuscular injection of tiletamine and zolazepam (4 mg ∙ kg^-1^; Zoetis, Parsippany, NJ), followed by an intracardiac injection of pentobarbital sodium (0.4 mL · kg^-1^; Schering-Plough, Union, NJ).

The study was designed to investigate the effects of a high-fructose high-fat diet and probiotic supplementation in the pathogenesis and progression of pediatric non-alcoholic fatty liver disease. Twenty-eight 13-d-old Iberian pigs were housed in pairs in 1.5 × 1.5 m pens, and randomly distributed to receive 1 of the 4 liquid diets for 10 consecutive weeks: 1) control (CON-N; n = 4 pens), 2) high-fructose high-fat (HFF-N; n = 3 pens), 3) CON + probiotics (CON-P; n = 4 pens), and 4) HFF-N + probiotics (HFF-P; n = 3 pens). All animals were euthanized on d 70 of the study, and tissue from left medial segment in the liver was collected and frozen in liquid nitrogen for metabolomic analysis.

Metabolomics assays were performed in 26 liver samples (8 CON-N, 8 CON-P, 5 HFF-N, 5 HFF-P) using protein precipitation extraction with ultra-performance liquid chromatography-tandem mass spectrometry. Metabolite intensities were normalized to that of internal standards added during the extraction and to sample weight to account for small variations in starting tissue. Data was expressed as peak areas under the curve. A total of 218 metabolites were detected in liver tissue, with a few missing values produced due to metabolite peaks not being detected. These measurements were set to missing by placing a dot (.) in the CSV file.

### Preliminary analysis

Before a model fitting to one-metabolite-at-a-time can be implemented with our ***%polynova_2way*** macro, data must be analyzed with multivariate statistics using software such as R prcomp() function [8], MetaboAnalyst or Primer-E (v.7; Primer-E Ltd., Plymouth, UK), as previously described [7]. Briefly, liver data was imported into Primer-E, log transformed into a normal distribution approximation and analyzed with a Euclidean distance matrix. A nonparametric permutational analysis of variance (PERMANOVA; Primer-E) with diet × probiotic as fixed effects, and pen nested in diet × probiotic as random effect, was used for testing the null hypothesis of no difference between groups under a reduced model, 9,999 permutations, and type III (partial) sum of squares. PERMANOVA compares groups (i.e. diets) of multivariate sample units (i.e. pigs) by constructing ANOVA-like test statistics from a matrix of resemblances (distances, dissimilarities, similarities) calculated among the sample units, and obtains P-values using random permutations of observations among the groups. Results are presented in Table 1.

**Table 1:** Permutational analysis of variance of liver metabolites showing a significant effect of diet, but no evidence for probiotic or probiotic × diet effects. PERMANOVA was run under a reduced model, 9,999 permutations, and type III (partial) sum of squares.

Item	df	SS	MS	Pseudo-F	P(perm)	Unique perms
Probiotic	1	2.598	2.598	0.489	0.905	9921
Diet	1	192.3	192.3	36.18	**0.0002**	9891
Probiotic × Diet	1	4.114	4.114	0.774	0.644	9931
Pen (Probiotic × Diet)	10	53.64	5.364			
Residual	12	57.42	4.785			
Total	25	322.7				

**Fig 1:**
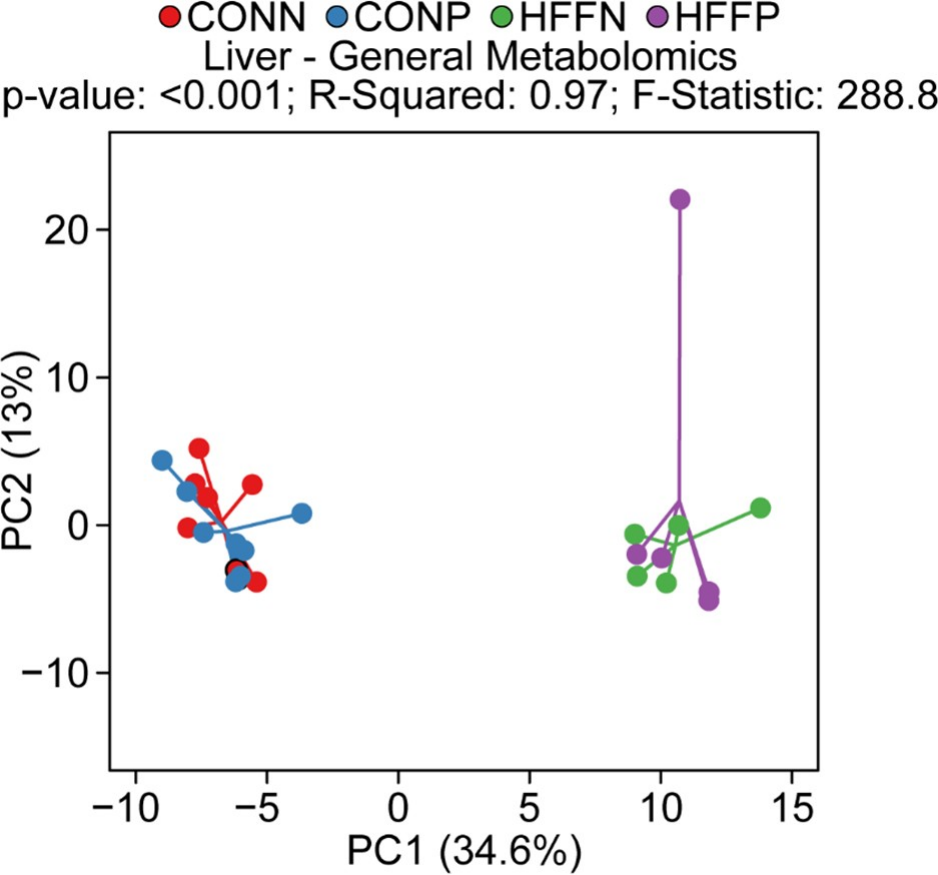
PCA analysis to visualize group discrimination in a 2-dimensional scores plot. Two-dimensional visualization of PCA scores are projected from their group centroid along components 1 and 2. P-value, R-squared, and F-statistic are derived from ANOVA assessed on first principal component. Each point represents an individual pig and color of point denotes diet. Consistent with PERMANOVA analysis, PCA showed a separation of HFF and CON samples, but not a probiotic effect.

Metabolomics data was further assessed by Principal Component Analysis (PCA) to visualize group discrimination in a 2-dimensional scores plot (Fig 1).

PCA analyses were conducted in the R Statistical Language using the prcomp() function [8]. Cube-root transformed concentrations or relative abundance data were scaled to unit variance prior to PCA assessment. PCA scores from components 1 and 2 were plotted and overlaid with diet and probiotic classifiers. Component 1 was assessed for group differences using ANOVA and we reported the total R^2^, F-statistic, and P-value from these models.

### The %polynova_2way SAS macro

**Fig 2:**
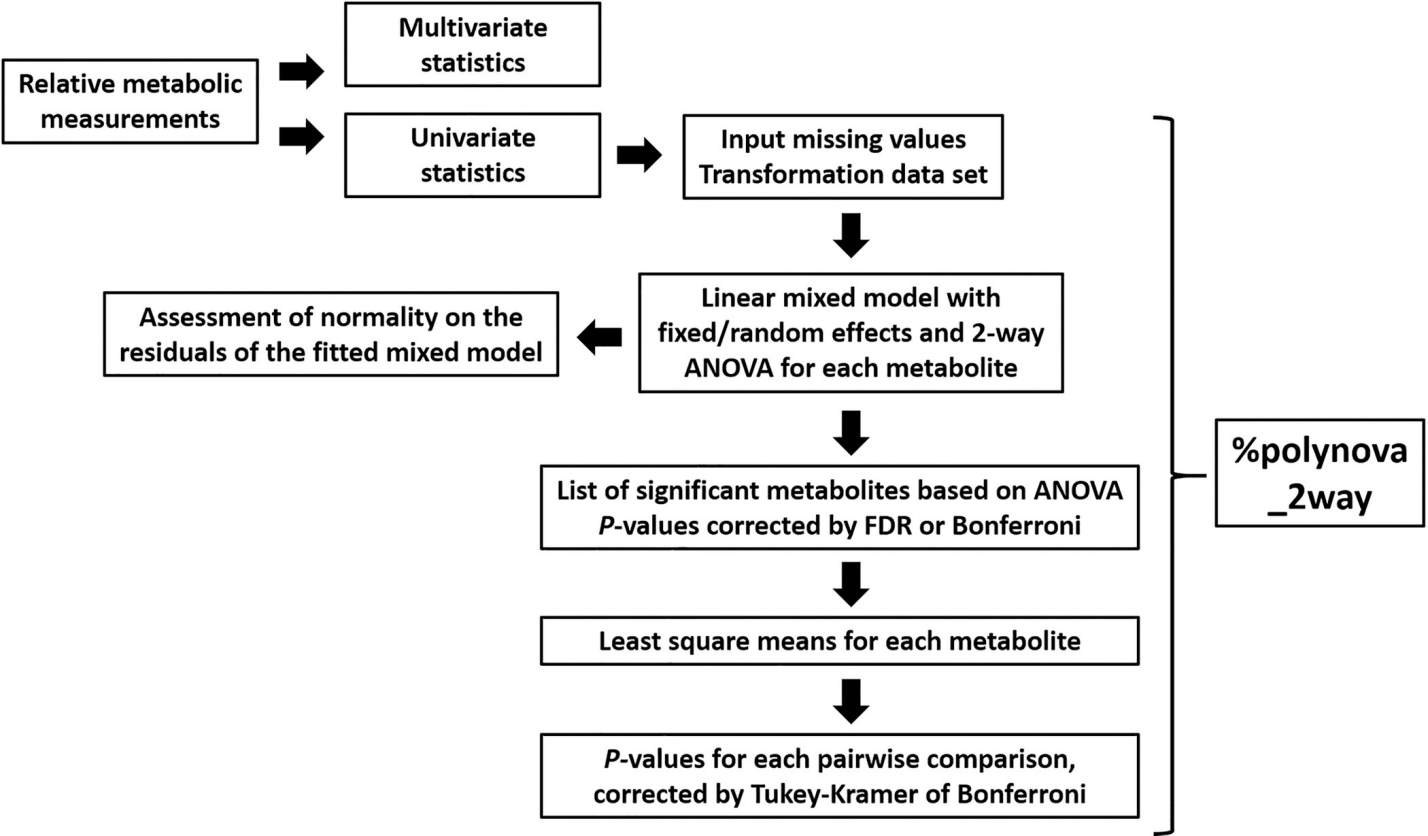
Overview of analytic workflow of *%polynova_2way* SAS macro. Input is the table of relative metabolic measurements. The ***%polynova_2way*** macro outputs a table in excel format that combines all the results to facilitate the identification of significant metabolites.

Our ***%polynova_2way*** macro, written in SAS software [9], allows for the identification of metabolites differentially expressed between treatment groups in complex study designs that involve treatment interactions and random effects. The SAS code is provided in S1 Code file. For each metabolite, the macro calculates the least squares means using a linear mixed model with fixed and random effects, runs a two-way ANOVA, adjusts the ANOVA P-values for the number of metabolites using the FDR [3] or Bonferroni [4] procedure, calculate the P-values of the least squares mean differences, and adjust these P-values for the number of pairwise comparisons using Tukey-Kramer or Bonferroni procedures. Finally, the macro combines the results into a single table (easily viewable in Microsoft Excel) that allows the researcher to easily sort through the metabolites and identify those significantly different among treatments (Fig 2).

The ***%polynova_2way*** macro is comprised of multiple sub-steps for fully processing and modeling metabolomics data: 1) The raw CSV data file is imported into SAS using PROC IMPORT. The macro assumes that the raw data file is in wide format with a separate column for each treatment and metabolite measurement and a separate row for each individual in the study (See S1 Data file). 2) The data are transposed into a long format so that later procedures can exploit the BY statement to analyze all of the data in a single PROC step. 3) The macro transforms the data to investigate model assumptions for each individual metabolite (see Results and Discussion). 4) The mixed model is fitted using PROC MIXED (output of the model, including least squares means, is saved in a corresponding dataset). 5) Tests for normality are performed on the residuals of the fitted models and corresponding P-values are saved. 6) P-values from the previously run PROC MIXED are FDR- or Bonferroni-adjusted for the number of metabolites using PROC MULTTEST. 7) Least squares mean estimates and confidence intervals back-transformed to the scale of the original data are computed and saved in a corresponding dataset. 8) P-values from least squares mean differences are corrected for the number of pairwise comparisons by Tukey-Kramer or Bonferroni and saved in a corresponding dataset. The remainder of this macro re-organizes and consolidates all of these results into a single, concise table of information. A separate, secondary macro (***%export_mixed_res***) exports the results of ***%polynova_2way*** in one of two ways: 1) one large, consolidated CSV file, or 2) a separate CSV file for each component of the results listed above.

In its current state the macro is robust and requires only minimal changes based on the dataset specified. As mentioned, the macro assumes that the data are in wide format (step one above). The ***%polynova_2way*** macro can accommodate many different sizes of dataset without adjustment. The user must provide the input and output parameters, and macro variable values as specified in Table 2. All parameters of the ***%polynova_2way*** macro are required. There are four variables defined above the macro which specify: 1) ***path***: desired location of SAS dataset created by the macro (a single, SAS dataset version of the original CSV file), 2) ***respath***: desired location of the resulting output, 3) ***normgraphpath***: desired location of the normality diagnostics, including normal quantile-quantile plots, and 4) ***fitgraphpath***: desired location of the fitted values versus residuals plots. The ***class_statement***, ***model_vars***, and ***random_statement*** parameters are all used in the respective PROC MIXED components: class statement, model statement, and random statement. These parameters give the user some flexibility to specify different types of models that PROC MIXED can accommodate. However, the remainder of the macro assumes that the user is fitting a model with two main effects plus an interaction that could include random effects. For more detail we refer the reader to the SAS documentation for PROC MIXED [9].

**Table 2:** Macro variables, input, and output parameters for *%polynova_2way* SAS macro.

Parameter	Description
**Input**	
path	Path to desired location of SAS dataset version of original CSV data file created by the macro. Do not include file name. e.g. C:\Users\manjarin\PLOSONE\dataset
respath	Path to desired results location, not including file name. e.g. C:\Users\manjarin\PLOSONE\dataset
normgraphpath	Path to normal quantile-quantile plots if requested. e.g. C:\Users\manjarin\PLOSONE\graphs
fitgraphpath	Path to fitted value plots if requested. e.g. C:\Users\manjarin\PLOSONE\graphs
raw_dsetin	Name of input dataset; Assumes this is path to CSV file, including file name C:\Users\manjarin\PLOSONE\dataset\S1_Data.csv
**Macro variables (%macro polynova_2way)**	
normtrans	Transformation for normality 0 (None), 1 (Log; default), 2 (Square root)
pval_adjust	Correction of ANOVA *P*-values by number of metabolites 1(FDR; default), 2(Bonferroni)
excludevars	Names of variables not to use as response, but used in model e.g. Trx1 Trx2 pig pen
ignorevars	Names of variables not used in model at all e.g. Sex
treatment_var1	Name of the first treatment variable in your dataset e.g. Trx1
treatment_var2	Name of the second treatment variable in your dataset e.g. Trx2
class_statement	Class variables in the PROC MIXED e.g. Trx1 Trx2 pen
model_vars	Fixed effects and interaction in the PROC MIXED e.g. Trx1|Trx2
random_statement	Random effects in PROC MIXED e.g. pen(Trx1*Trx2)
pairwise_adjust	Correction of P-values of least squares mean differences by number of pair-wise comparisons. For comparisons of marginal means (main effects), use Tukey-Kramer. For comparisons of cell means (interaction), use Bonferroni (see Results and Discussion section for additional information) Tukey-Kramer (default), Bonferroni
**Output labels (%*export_mixed_res*)**	
name	Name of output CSV dataset file without “.csv” e.g. Liver_PLOS
trans	Name of transformation applied e.g. Log
adjust	ANOVA P-value correction e.g. FDR
singlefile	TRUE specifies all results should be consolidated and exported in a single CSV file TRUE (default) or FALSE

Use of the macro does require a few precautions. First, SAS maintains a number of restrictions on variable names (metabolite names in the applications presented here). Before applying the macro to a dataset all of the variable names should conform to these restrictions, most importantly that they are less than 32 characters long and do not contain special characters other than underscores. Second, file names and file paths specified as parameters of the macro cannot contain spaces. Finally, the results are currently sorted by the first treatment variable’s P-values. So, the user should keep this in mind in determining which column they want to make the first treatment variable.

## Results and discussion

We demonstrate the use of the ***%polynova_2way*** macro in the analysis of metabolomics data generated in our pig study to investigate the role of HFF diets and probiotics in pediatric NAFLD. The dataset used in this example included pig identification number, pen, sex, Trx1 (probiotic) and Trx2 (diet), followed by a list of all the metabolites analyzed in liver tissue. In particular, the statistical model being used here is

$${\text{Y}}_{\text{ijkl}}=\mu +\alpha_{\text{k}}+\gamma_{\text{l}}+{\left(\alpha \gamma \right)}_{\text{kl}}+{\text{b}}_{\text{j}}{\left(\alpha \gamma \right)}_{\text{kl}}+{\text{e}}_{\text{ijkl}}$$

where Y_ijkl_ is the metabolite measured for pig *i* in pen *j* within diet level *k* and probiotic level *l*, *μ* is the intercept, *α*_k_ is the fixed effect of the *k*^th^ level of diet, γ_l_ is the fixed effect of the *l*^th^ level of probiotic, (*α*γ)_kl_ is the fixed effect of the interaction between diet level *k* and probiotic level *l*, b_j_(*α*γ)_kl_ is the random effect of the pen nested in diet × probiotic, and e_ijkl_ is the experimental error for pig *i*. We assume that b_j_, the random effects of pen, are normally distributed with zero mean and some unknown covariance matrix. Note that this model will get fit for each metabolite (Y) one-by-one and is accomplished in SAS with the use of the BY statement below. The corresponding PROC MIXED syntax for this model is the following:

proc mixed data = metabolitedata;
by Response;
class Trx1 Trx2 pen;
model Value = Trx1|Trx2/ddfm = KENWARDROGER outpm = resids residual;
random pen(Trx1*Trx2);
lsmeans Trx2|Trx2/adjust = tukey cl;
run;

The SAS output consists of a single horizontal table in an excel file that contains results for each metabolite organized in rows, and the parameters from the SAS output in columns. To fit the table in the result section of the manuscript, it has been subdivided in 4 smaller tables presented below (Tables 3–6). Information included in the SAS output presented in Table 3 includes: 1) response variable, 2) results from the Shapiro-Wilk and Kolmogorov–Smirnov tests of normality conducted on residuals, 3) results of the 2-way ANOVA expressed as P-values and FDR- or Bonferroni corrected P-values for Trx1, Trx2 and their interaction (inter).

**Table 3:** Output of analysis for implementing the *%polynova_2way* macro (Part 1/4).

Response	Transformation	Shapiro_Wilk	Kolmogorov	Pvalue_Trx1	FDR Pvalue_Trx1	Pvalue_Trx2	FDR Pvalue_Trx2	Pvalue_Inter	FDR Pvalue_Inter
pyridoxate	log	0.54	0.15	0.66	0.99	0.001	0.001	0.15	0.95
acetylchol	log	0.55	0.15	0.94	0.98	0.008	0.01	0.87	0.95
adenosine	log	0.72	0.15	0.05	0.98	0.05	0.05	0.30	0.95
anthranilic	log	0.65	0.15	0.59	0.98	0.01	0.01	0.90	0.89

**Table 4:** Output of analysis for implementing the *%polynova_2way* macro showing least square means and confidence intervals for Trx 1 (Part 2/4).

Response	LS_N	CI_Lower_N	CI_Upper_N	LS_P	CI_Lower_P	CI_Upper_P
pyridoxate	0.22	0.18	0.27	0.21	0.17	0.26
acetylchol	68154	52212	88965	69049	52897	90133
adenosine	2.31×10^8^	2.10×10^8^	2.53×10^8^	2.65×10^8^	2.40×10^8^	2.92×10^8^
anthranilic	29050	26594	31732	29962	27430	32729

**Table 5:** Output of analysis for implementing the *%polynova_2way* macro (Part 3/4).

Response	P_valueD Trx1 N_P	P_valueD Trx2 CON_HFF	P_valueD Trx1_Trx2 N_CON P_CON	P_valueD Trx1_Trx2 N_CON N_HFF	P_valueD Trx1_Trx2 N_CON _PHFF	P_valueD Trx1_Trx2 N_HFF P_CON	P_valueD Trx1_Trx2 P_CON P_HFF	P_valueD Trx1_Trx2 N_HFF P_HFF
pyridoxate	0.67	0.0007	0.41	0.09	0.005	0.02	0.0008	0.23
acetylchol	0.94	0.008	0.94	0.04	0.06	0.05	0.07	0.88
adenosine	0.04	0.05	0.35	0.04	0.96	0.009	0.47	0.06
anthranilic	0.59	0.01	0.74	0.04	0.09	0.02	0.05	0.67

**Table 6:** Output of analysis for implementing the *%polynova_2way* macro (Part 4/4).

Response	TukeyD Trx1 NP	TukeyD Trx2 CON HFF	TukeyD Trx1_Trx2 N_CON P_CON	TukeyD Trx1_Trx2 N_CON N_HFF	TukeyD Trx1_Trx2 N_CON _P_HFF	TukeyD Trx1_Trx2 N_HFF P_CON	TukeyD Trx1_Trx2 P_CON P_HFF	TukeyD Trx1_Trx2 N_HFF P_HFF
pyridoxate	0.67	0.001	0.83	0.32	0.02	0.09	0.004	0.61
acetylchol	0.94	0.008	1.00	0.16	0.22	0.18	0.24	0.99
adenosine	0.04	0.05	0.75	0.13	1.00	0.03	0.87	0.22
anthranilic	0.60	0.009	0.99	0.14	0.28	0.09	0.19	0.97

In addition to Shapiro-Wilk and Kolmogorov-Smirnov tests, normality diagnostics including quantile-quantile and fitted values vs. residuals plots are also generated in an additional pdf file. The decision to implement a transformation of the data or not needs to be made on a metabolite-by-metabolite basis, especially since mixed models will be fitted to one-metabolite-at-a-time. In fact, if transformation is applied indiscriminately to metabolites for which it is not needed, the transformation is likely to distort distributional assumptions and create problems where none were present. Therefore, the normality diagnostic output in the macro must be used along with the function **normtrans = 0 (not-transformed), 1 (log-transformed), 2 (square-root transformed)** to determine which metabolites need to be transformed, and which transformation need to be implemented, before examining the PROC MIXED output. The ***%polynova_2way*** macro has 2 limitations. The macro cannot transform individual metabolites within the dataset, so once non-normally distributed metabolites have been identified, the user will have to individually transform them in the csv file, and then rerun the macro with the function **normtrans = 0**. In addition, the macro can only implement log- and square root-transformations of the data. If different transformations are needed, the user will have to investigate them with methods such as Box-Cox. A SAS macro for Box-Cox that utilizes PROC MIXED has been published by Piepho [10].

An important output in Table 3 are the P-values and corrected P-values of the ANOVA for each metabolite, which allows the researcher: 1) to select significant metabolites for each factor and their interaction, and 2) to correct the P-values for the total number of metabolites using the FDR or Bonferroni procedure. For example, in our liver dataset the multivariate analysis (Table 1) identified a significant effect for diet, but not for probiotics or diet × probiotics, so we used Trx2 FDR-corrected P-values to select significant metabolites between CON and HFF diets.

To describe the expected magnitude of treatment effects, the SAS output also includes the least square means estimates (marginal means) and confidence intervals (Table 4). If the data were transformed for analysis, least squares means are back-transformed to the scale of the original data for reporting purposes.

Tables 5 and 6 consist of raw P-values and corrected P-values of the least squares mean differences for each metabolite, which allows the researcher: 1) to investigate the level of significance for each pairwise comparison, and 2) to correct the P-values for the number of pairwise comparisons by using Tukey-Kramer or Bonferroni procedures. In our liver dataset, given that we are only interested in the effect of diet, we used Trx2 CON_HFF Tukey-adjusted P-values to investigate differences between CON and HFF diets. Of note, Tukey-Kramer’s adjustment for multiplicity is appropriate to control Type I error when main effects are significant and the pairwise comparisons of interest are between marginal treatment means (i.e. N vs P, or CON vs HFF). This is indeed the case for the example explored herein. However, when interactions are significant and pairwise comparisons of interest need to be made between treatment cell means (i.e. simple effects), Tukey-Kramer’s adjustment quickly starts to overpenalize due to multiple unnecessary tests. Instead, relevant simple effects to follow significant interactions should be adjusted using a Bonferroni adjustment based on the number of comparisons of interest [11].

Strengths of the **%polynova_2way** SAS macro include its ability to statistically analyze group differences in metabolites in study designs with a factorial treatment structure and hierarchical design structure, and subsequently package an assortment of results into a single Excel. A limitation of the macro is that before it can be implemented, the user must investigate the data using multivariate statistics that needs to be run in additional software such as Primer-e, MetaboAnalyst or R language.

In conclusion, this paper presents a flexible SAS macro for analysis of metabolomic studies with two-way factorial treatment structures and experimental design leading to structured data. The macro represents a powerful tool that allows researchers to incorporate both fixed and random effects in the statistical models, implement transformation of the data to improve normality, select significant metabolites using FDR or Bonferroni corrections, and adjust P-values for multiple pairwise comparisons. The macro described herein increases the capacity of metabolomics researchers to appropriately analyze complex study designs by streamlining the implementation of sophisticated methods for large data sets. Because we acknowledge the focus on two-way analyses could be limiting, we have included code for a second macro, **%polynova_1way** which performs similar one-way analyses.

## Supporting information

S1 Data Sample liver dataset used to demonstrate the functioning of the *%polynova_2way* SAS macro. (CSV)

S1 Code The *%polynova_2way* source code to read in the data and run the macro. (SAS)

S2 Code The *%polynova_1way* source code to perform 1-factor ANOVA. (SAS)
